# PRISMA 2020: strengthening transparency and usefulness of systematic reviews

**DOI:** 10.1016/j.bjane.2026.844834

**Published:** 2026-09-20

**Authors:** Leonardo Roever, Matthew J. Page, David Moher

**Affiliations:** a Brazilian Evidence Based Health Network, Department of Clinical Research, Uberlândia, MG, Brazil; b Monash University, School of Public Health and Preventive Medicine, Methods in Evidence Synthesis Unit, Melbourne, Australia; c Ottawa Hospital Research Institute, Centre for Journalology, Methodological and Implementation Research Program, Ottawa, Canada; d University of Ottawa, Faculty of Medicine, School of Epidemiology and Public Health, Ottawa, Canada; e University of Toronto, Dalla Lana School of Public Health, Institute of Health Policy, Management & Evaluation, Toronto, Canada

We are currently living in an era of unprecedented expansion of scientific literature. Millions of articles are published annually, making it virtually impossible for clinicians, policymakers, researchers, patients, and other stakeholders to keep up with all the available evidence. Systematic reviews are essential tools for organizing and interpreting this growing volume of information.^1^^,^^2^

However, the increasing popularity of systematic reviews has also brought significant challenges. Unnecessary duplicate reviews, opaque methods, incomplete search strategies, and inconsistent reporting can compromise the reliability of the synthesized evidence. In many cases, the absence of methodological details prevents the reproducibility of reviews, limiting their scientific value. Robust reporting guidelines are essential to improve the completeness, transparency, and reproducibility of systematic review reporting. However, transparent reporting alone does not ensure methodological rigor or validity of the review findings. PRISMA should be used in conjunction with tools that address the conduct of the review, risk of bias, certainty of evidence, and methodological assessment.^1^, ^2^, ^3^, ^4^

In this context, the PRISMA 2020 guidelines (Preferred Reporting Items for Systematic Reviews and Meta-Analyses) represent a fundamental milestone in strengthening the reliability of systematic reviews in contemporary healthcare.^1^, ^2^, ^3^, ^4^, ^5^ Figure 1 shows the functional structure of PRISMA.

**Figure 1:**
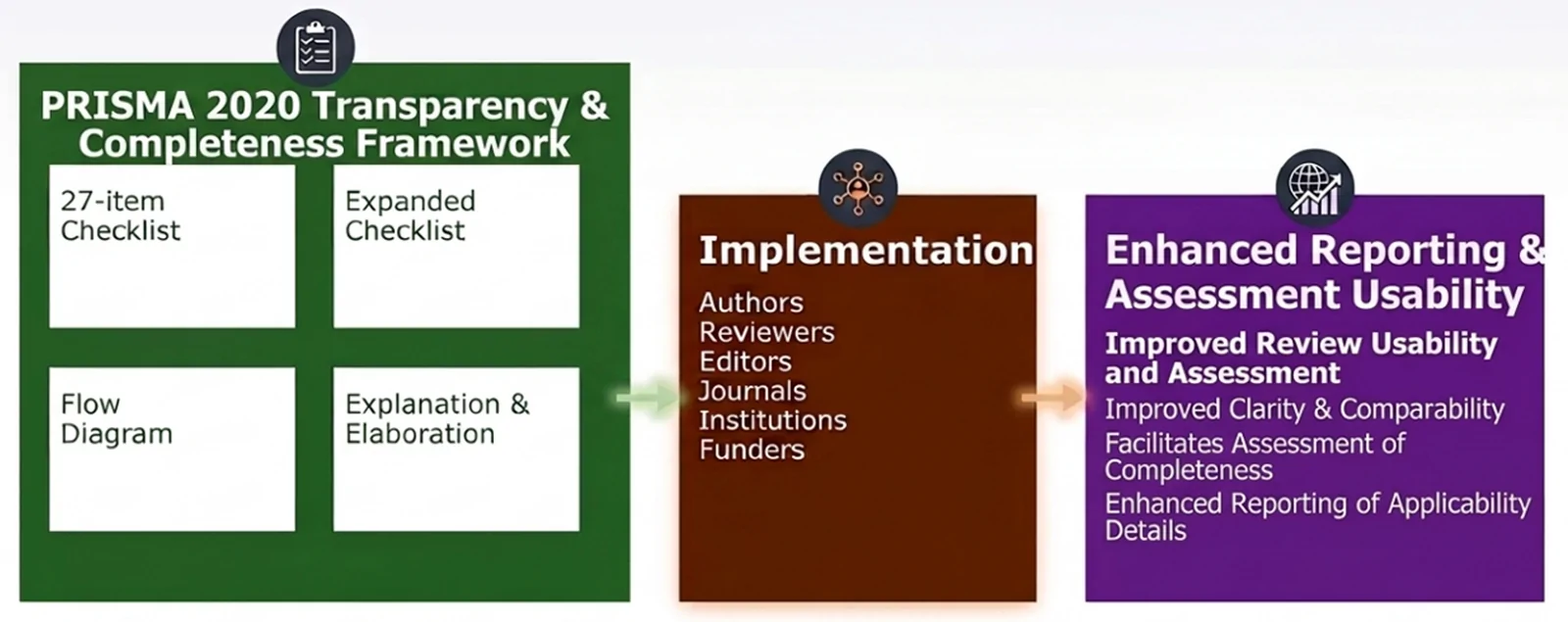
Shows the functional structure of PRISMA. The PRISMA 2020 framework (checklist, flow diagram, and explanation & elaboration document) provides a standardized structure that supports authors, editors, and reviewers across various institutions. Its primary goal is to enhance the clarity, completeness, and transparency of systematic review reports. By using this framework, stakeholder’s can improves the usability and assessability of evidence syntheses, enabling more rigorous and informed evaluation by guideline developers, clinicians, and policymakers, ultimately contributing to a more robust global health evidence base thorough complete and transparent reporting.

## Core components of PRISMA 2020 and their contribution to the transparency of systematic reviews

PRISMA 2020 represents the most recent update to the international PRISMA guidelines for reporting systematic reviews. Developed to reflect methodological advances of the preceding decade, PRISMA 2020 offers a structured set of items aimed at improving the transparency, completeness, and reproducibility of systematic reviews.^1^, ^2^, ^3^, ^4^, ^5^ There are also many PRISMA extensions providing reporting guidance for different types or aspects of systematic reviews [e.g., for reporting a protocol of a systematic review (PRISMA-P), a scoping review (PRISMA-ScR), and a systematic review with network meta-analysis (PRISMA-NMA)]. All PRISMA documents (e.g., checklist, explanation and elaboration paper for PRISMA 2020 and PRISMA extensions) can be accessed on the PRISMA website (https://www.prisma-statement.org/), which receives more than 200,000 site visits per month. PRISMA 2020 has been translated into 17 languages [including in Brazilian Portuguese and Spanish (see all translated documents at https://www.prisma-statement.org/translations)].

The PRISMA 2020 guideline consists of four main components:

1. The main checklist with 27 items and abstracts checklist with 12 items.
2. An expanded checklist (which includes detailed reporting recommendations for each item).
3. Flow diagram templates for authors to document the flow of documents through the study selection process.
4. The Explanation and Elaboration document (which provides exemplars of good reporting and explanations to enhance the use and understanding of PRISMA 2020).

These components were developed to guide authors, peer reviewers, and editors through all stages of communicating systematic reviews.

## The 27-item checklist

The core of PRISMA 2020 is the 27-item checklist, which defines the critical aspects that should be reported in a systematic review. These items cover all sections of a systematic review manuscript, from the title to the declarations (e.g., funding and competing interests) statements. These steps ensure that critical information about methods and results is presented clearly and completely.^1^

The items are organized into seven major sections of the scientific article:

1. Title: The title should explicitly identify the study as a systematic review. This identification facilitates indexing in databases and improves literature retrievability.
2. Abstract: The abstract should provide a structured description of the review's objectives, methods, results, and conclusions, allowing readers to quickly understand the nature and relevance of the study.
3. Introduction: In this section, authors should explain the rationale for the review in the context of existing knowledge and clearly define the review objectives or questions.
4. Methods: The methods section is one of the most detailed components of the checklist. It includes items related to:
  • Eligibility criteria for studies;
  • Information sources;
  • Search strategies;
  • Study selection process;
  • Data collection process;
  • Data items;
  • Study risk of bias assessment process;
  • Effect measures;
  • Synthesis methods;
  • Reporting bias assessment;
  • Certainty assessment.
5. Results
Authors should report: The number of studies identified, evaluated, and included; Characteristics of the selected studies; Study risk of bias assessments; Results of syntheses; Reporting bias assessments; Certainty assessments.
6. Discussion: The discussion should interpret the results in light of the existing literature, discuss limitations of the evidence included and the review processes used, and consider implications for clinical practice, health policies, and future research.
7. Other Information
The checklist also includes items related to: Registration and protocol for the review; Support (e.g., Funding) for the review; Competing interests of review authors; Availability of data, code and other materials.

These elements reinforce transparency and scientific integrity.

## The expanded checklist

In addition to the main list of 27 items, PRISMA 2020 provides an expanded checklist, which offers more detailed guidance on how each item should be reported.^1^ The goal of the expanded checklist is to reduce ambiguities in the interpretation of checklist items, helping authors to fully understand the expected level of detail.

## The PRISMA Flow diagram

Another central component of PRISMA 2020 is the study selection flow diagram, which provides a visual representation of the process of identifying, screening, and inclusion of studies. This visual representation significantly improves the transparency of the study selection process, allowing readers to clearly understand how evidence was identified and filtered. Different flow diagram templates are available depending on whether the review is new or updated, and on the type of sources searched.

## Explanation and elaboration paper

The Explanation and Elaboration paper provides an in-depth description of each item on the checklist.^1^ This document includes: The scientific rationale for each item; Exemplars of reporting each checklist item drawn from published systematic reviews; Detailed guidance on good reporting practices.

The aim of this document is to help authors understand not only what should be reported, but also why each piece of information is important for the interpretation of the systematic reviews.

## Importance of PRISMA 2020

The importance of robust reporting guidelines has become even more evident in an era characterized by the rapid dissemination of scientific information and misinformation. Spurious associations between clinical interventions and health outcomes can spread rapidly when based on poorly conducted or poorly reported evidence. The increasing integration of Artificial Intelligence (AI) into evidence synthesis offers important opportunities to improve the efficiency of selected tasks, including literature screening, citation management, and data extraction. However, AI-assisted technologies should be regarded as supportive tools rather than replacements for scientific judgment. Critical stages of the systematic reviews process ‒ including study selection, critical appraisal, interpretation of findings, assessment of the certainty of the evidence, and formulation of conclusions ‒ require human expertise, methodological competence, and accountability. Accordingly, the use of AI should always be accompanied by appropriate human oversight and independent verification of outputs. To promote transparency and reproducibility, authors should explicitly report the AI tools used, their specific purpose within the review workflow, the extent of human supervision, the procedures adopted to verify AI-generated outputs, and the measures taken to preserve an auditable record of the research process. Although PRISMA 2020 was developed before the widespread adoption of generative AI, its core principles of transparency, completeness, and reproducibility provide a strong foundation upon which future reporting guidance for AI-assisted evidence synthesis can be built.^4^, ^5^, ^6^

Although systematic reviews are often seen as methodological tools, their impact extends far beyond academia. They inform clinical guidelines, regulatory decisions, intervention funding, patient decision-making, and global health strategies. When properly reported, systematic reviews can help identify evidence gaps, underrepresented populations, and inequalities in scientific output. In this way, guidelines such as PRISMA 2020 also contribute to promoting greater equity in global health research. By improving the transparency of evidence syntheses, PRISMA 2020 allows researchers, funders, and policymakers to better understand where the evidence is strong and where it is still insufficient.

PRISMA 2020 represents an evolution in the reporting of systematic reviews and meta-analyses. At a historical moment when trust in science faces challenges and the volume of scientific information grows exponentially, robust standards of transparency and reporting are essential to preserving the credibility of health evidence. The true impact of PRISMA 2020, however, will depend on the collective commitment of researchers, reviewers, editors, and funders to fully adopt it. Only in this way will it be possible to ensure that systematic reviews continue to play their central role in promoting evidence-based decisions and, ultimately, in improving the health of populations worldwide.

To maximize the impact of PRISMA 2020 authors, peer reviewers, and journal editors should work collaboratively to strengthen the transparency and reproducibility of systematic reviews. Authors should prospectively register their systematic review protocols and transparently report and justify any deviations from the original protocol. Complete and reproducible search strategies for all information sources should be provided, together with transparent descriptions of duplicate screening, study selection, and data extraction procedures. Systematic reviews should comprehensively report risk-of-bias assessments and the certainty of the evidence using established frameworks, such as GRADE, whenever appropriate. In keeping with policy frameworks in several parts of the world, there is an increasing expectation for data sharing to occur responsibly, where ethical, legal, and commercial obligations allow, in line with FAIR principles: as open as possible, as closed as necessary. Authors of systematic reviews should make extracted datasets, analytic code, statistical scripts, and supplementary materials publicly available to facilitate verification and reproducibility. Finally, as Artificial Intelligence (AI) becomes increasingly integrated into evidence synthesis workflows, any use of AI-assisted technologies should be explicitly disclosed, together with appropriate human oversight. Collectively, these practices reinforce the principles of transparency, reproducibility, and research integrity that underpin PRISMA 2020 and enhance the reliability of systematic reviews for clinical decision-making.

## Use of generative AI tools

During the preparation of this manuscript, the authors used ChatGPT 5 (OpenAI, San Francisco, CA, USA) solely to improve language clarity and readability. No AI tool was used to generate scientific conclusions, select references, assess evidence, analyze data, or interpret findings. The authors reviewed and edited all AI-assisted output and take full responsibility for the final content.

## Data availability statement

No new data were created or analyzed in this article. Data sharing is not applicable to this article.

## Authors’ contributions

Leonardo Roever: Writing-original draft; writing-revision and editing.

Matthew J. Page: Writing-revision and editing; writing-original draft.

David Moher: Writing-original draft; writing-revision and editing; conceptualization.

## Funding

The authors declare that they did not receive any financial support for this work and/or its publication.

## Conflicts of interest

Matthew J. Page and David Moher are authors of the PRISMA 2020 Statement. This role is disclosed for transparency. The authors declare that this work was carried out in the absence of any commercial or financial relationships that could be interpreted as a potential conflict of interest.
